# Nutritional Quality, Sensory Analysis and Shelf Life Stability of Yogurts Containing Inulin-Type Fructans and Winery Byproducts for Sustainable Health

**DOI:** 10.3390/foods9091199

**Published:** 2020-08-31

**Authors:** Maite Iriondo-DeHond, José Manuel Blázquez-Duff, María Dolores del Castillo, Eugenio Miguel

**Affiliations:** 1 Instituto Madrileño de Investigación y Desarrollo Rural, Agrario y Alimentario (IMIDRA), N-II km 38,200, 28800 Alcalá de Henares, Spain; maite.iriondo@madrid.org (M.I.-D.); jmbd_1994@hotmail.com (J.M.B.-D.); eugenio.miguel@madrid.org (E.M.); 2 Instituto de Investigación en Ciencias de la Alimentación (CIAL) (CSIC-UAM), C/ Nicolás Cabrera, 9, 28049 Madrid, Spain

**Keywords:** α-glucosidase inhibition, inulin-type fructans, yogurts, sustainable health, winery byproducts

## Abstract

The aim of the present study was to evaluate the use of winery byproduct extracts (grape pomace, seed and skin) and a mixture of inulin-type fructans (inulin and FOS) as suitable ingredients for the development of yogurts with antioxidant and antidiabetic properties. Their effect on the physicochemical, textural, microbiological and sensory parameters of yogurts was evaluated during 21 days of refrigerated storage. The incorporation of winery byproduct extracts in yogurt resulted in a significant increase (*p* < 0.05) in total phenolic content (TPC) and antioxidant and antidiabetic properties, compared to the controls. The grape skin yogurt showed the highest (*p* < 0.05) TPC (0.09 ± 0.00 mg GAE/g yogurt) and antioxidant capacity (7.69 ± 1.15 mmol TE/g yogurt). Moreover, the grape skin yogurt presented the highest (*p* < 0.05) inhibition of the activity of the enzyme α-glucosidase (56.46 ± 2.31%). The addition of inulin-type fructans did not significantly (*p* > 0.05) modify the overall antioxidant capacity or inhibition of the enzyme α-glucosidase of control and winery byproduct extract yogurts. Yogurts containing winery byproduct extracts and dietary fiber achieved high overall acceptance scores (6.33–6.67) and showed stable physicochemical, textural and microbiological characteristics during storage, assuring an optimal 21-day shelf life. According to their antioxidant and antidiabetic properties, we propose the yogurt containing grape skin extract, together with inulin and FOS, as a novel food product for the promotion of sustainable health.

## 1. Introduction

Sustainable health is defined as the promotion of healthy ageing by preventing the risk of diseases [1]. Diet-related cardiometabolic diseases pose a substantial health and economic burden, causing 17 million deaths worldwide [2]. Consequently, there is an increasing demand for foods that promote sustainable health and well-being. 

Different strategies for the promotion of healthier foods include product reformulation with a reduction of critical nutrients, such as added sugars, sodium and saturated fatty acids. Sweetened dairy products stand as one of the major food categories to focus action on, due to their excessive amount of added sugars [3]. The incorporation of inulin-type fructans, such as inulin and fructo-oligosaccharides (FOS), in dairy foods may help replace their sugar content. Inulin has been described to improve dairy products’ texture, whereas FOS has been previously applied for its sweetening properties [4,5]. Moreover, inulin consumption has been associated with several health benefits, including reduced incidence of diabetes and obesity due to their influence on appetite and energy intake through various mechanisms, including production of short-chain fatty acids (SCFA) due to colonic fermentation and subsequent regulation of gut hormones [6]. In addition, the incorporation of inulin-type fructans in yogurt may help consumers reach the 25 g of dietary fiber per day recommendation established by the European Food Safety Authority (EFSA). Similarly, food fortification with bioactive compounds, such as polyphenols, is another strategy for the incorporation of health-promoting ingredients in the human diet. 

The wine-making industry produces a vast amount of byproducts which are well-known for their antioxidant and antidiabetic properties [7]. Their use as ingredients in foods increases the sustainability of the food chain and could exert health-promoting effects through bioactivity beyond the basic nutrient composition. In this sense, the antidiabetic properties of the winery ingredients incorporated into a complex dairy matrix, such as yogurt, have not been previously studied. Therefore, the aim of this study was to evaluate the use of grape pomace, grape seed, grape skin, inulin and FOS as suitable ingredients for the development of yogurts with antioxidant and antidiabetic properties. In addition, the effects of these ingredients on the physicochemical, textural, microbiological and sensory parameters of yogurts were evaluated during a 21-day refrigerated storage.

## 2. Materials and Methods 

### 2.1. Raw Materials

Ingredients used for yogurt preparation included ultra-high temperature (UHT) whole cow milk (Pascual, Madrid, Spain), inulin Orafti^®^GR and fructo-oligosaccharides Orafti^®^P95 (Beneo, Leuven, Belgium) and yogurt starter culture YO-MIX 300 (Danisco DuPont, Brabrand, Denmark) containing *Streptococcus thermophilus* and *Lactobacillus delbrueckii* subsp. *bulgaricus.*

### 2.2. Extracts

Food-grade commercial extracts from winery byproducts (grape pomace, seed and skin) were purchased from Natac (Madrid, Spain). Grape pomace extract was composed of the entire fruit, including grape skin and seeds. 

### 2.3. Yogurt Samples

Eight set-type yogurt formulations were prepared, combining the three winery byproduct extracts (grape pomace, seed and skin) with and without inulin-type fructans (Y-IGP, Y-IS, Y-ISK and Y-GP, Y-S, Y SK; respectively), a control with inulin-type fructans and no winery byproduct extracts (Y-IC), and a control without inulin-type fructans nor winery byproduct extracts (Y-C). Yogurt samples were produced by heating the milk up to 45 °C, to inoculate the lyophilized starter culture (45 mg starter culture/L of milk). Then, a mixture of inulin (7 g/100 mL) and FOS (10 g/100 mL) was added. Winery byproduct extracts were dissolved in water and added to a concentration of 5 mg/mL in the milk. In control yogurts, the corresponding amount of water was added without extract. Milk was stirred, separated into pots and incubated at 45 °C for 5 h, which was the time needed for the yogurts to reach a pH value of approximately 4.6. Samples were stored at 4 °C, until further analyses. Yogurts were prepared in triplicate, in three independent sessions. 

We studied the isolated effect of inulin-type fructan addition on the health-promoting properties of the yogurts containing winery byproduct extracts (Y-IC, Y-IGP, Y-IS, Y-ISK and YC, Y-GP, Y-S, Y SK). Composition and technological analyses were performed on yogurts containing both ingredients (Y-IC, Y-IGP, Y-IS, Y-ISK). 

### 2.4. Sample Preparation

Prior to composition (Section 2.5) and health-promoting (Section 2.6) analyses, the extract and yogurt samples were treated as follows:

#### 2.4.1. Extracts

Grape pomace, seed and skin extracts were diluted in a dimethyl sulfoxide/ water mixture (1:10), at a final concentration of 1 mg/mL, and filtered with a 0.45 µm filter (Minisart Sterile16555, Merck, Darmstadt, Germany). Samples were stored at −20 °C, until composition and health-promoting activity analyses.

#### 2.4.2. Yogurts

On day 1, 7, 14 and 21, yogurt samples (10 g) were diluted with 10 mL of distilled water and filtered in two steps with a Whatman filter paper grade 1, followed by a 0.45 µm filter (Minisart Sterile16555, Merck, Darmstadt, Germany). The filtered extracts were stored at −20 °C, until composition and health-promoting analyses were performed.

### 2.5. Composition Analyses 

Folin–Ciocalteu adapted to a micro-method format was used for the analysis of total phenolic content (TPC) in winery byproduct extracts and yogurt samples [8]. A gallic acid calibration curve (0.01–1 mg/mL) was used for quantification. Measurements were performed in triplicate.

In yogurts, total protein was determined by the Kjeldahl method, as defined in Commission Regulation No. 152/2009. Total fat was calculated by gravimetry, as defined in Commission Regulation No. 152/2009. Fatty acid profile was obtained by gas chromatography (Agilent 7820A GC System equipped with Flame Ionization Detector) analyses, calculated according to the ISO 12966-2:2017. Lactose was measured by using the Lactose-D-galactose Assay Kit (Megazyme, Wicklow, Ireland).

### 2.6. Health-Promoting Properties

#### 2.6.1. Antioxidant Capacity

● *2,2′-azino-bis (3-ethylbenzothiazoline-6-sulphonic acid) (ABTS) assay*

The ABTS decolorization assay was performed as described in reference [9]. Aqueous solutions of Trolox (0.15–2.0 mM) were used for calibration. Measurements were performed in triplicate.

● *Oxygen Radical Absorbance Capacity (ORAC) assay*

The ORAC assay was applied [10] with modifications [11]. Measurements were performed in triplicate.

#### 2.6.2. Antidiabetic Properties

α-Glucosidase inhibition was analyzed [12,13], with modifications [14]. Results were expressed as the concentration causing 50% inhibition (IC50) for winery byproduct extracts and as percentage of α-glucosidase inhibition for yogurt samples. All measurements were performed in triplicate.

### 2.7. Technological and Shelf-Life Characterization 

The technological characterization (physicochemical and microbiological parameters) of yogurts containing winery byproduct extracts and inulin-type fructans was used as an indicator of the general quality of the yogurt during a 21-day shelf-life period. 

#### 2.7.1. Physicochemical Parameters

Moisture content was determined as described in AOAC-925.10. Yogurt’s pH was measured with a Hanna Instruments HI5521 pH meter. Titratable acidity was determined according to ISO/TS 11869, IDF:RM 150 and expressed as g lactic acid/100 g of yogurt. 

Yogurt syneresis was calculated using a centrifugation method [15] and expressed in percentage, according to the following equation:

Syneresis (%) = [expelled whey (g)/yogurt mass (g)] × 100(1)

The textural parameters of yogurts were obtained in triplicate, using a TA.XTplus Texture Analyzer and analyzed with the Exponent software (Stable Micro Systems, UK). A back-extrusion test was carried out, using a cylindrical stainless-steel probe (35 mm diameter) which was pressed into a cylindrical container (50 mm in diameter and 50 mm high). The probe penetrated the sample to a depth of 10 mm at 1 mm/s. Firmness (N) and consistency (Ns) were calculated from the deformation curves.

#### 2.7.2. Viability of Starter Bacteria

Bacterial counts were carried out in triplicate, by following the colony count technique (ISO 7889-IDF 117). *L. bulgaricus* colonies were counted in Man Rogosa Sharpe (MRS) agar (Prodinasa, Hispanlab SA, Madrid, Spain) after aerobic incubation at 37 °C for 72 h. *S. thermophilus* colonies were counted in M17 agar (Prodinasa, Hispanlab SA, Madrid, Spain) after aerobic incubation at 37 °C for 48 h. Results were expressed as log CFU/g of yogurt.

### 2.8. Pilot Consumer Analysis

The pilot consumer hedonic test (*n* = 30) was performed in panel booths conforming to international standards (ISO 8589:2007). Yogurts (30 mL) were coded with a three-digit number and presented in random order, to prevent first-order and flavor carryover effects. Consumers were asked to rate the appearance, odor, texture, flavor and overall acceptability of yogurts, using a 9-point hedonic scale (9 = like extremely, and 1 = dislike extremely). 

### 2.9. Statistical Analysis

Analyses were performed by using a one-way ANOVA with Tukey’s test for assessing differences between samples. A two-way repeated measures ANOVA with Tukey’s test was used to analyze shelf-life data in different time points. Calculations were performed using IBM SPSS Statistics version 24.

## 3. Results and Discussion

### 3.1. Composition and Health-Promoting Properties of Winery Byproduct Extracts 

TPC analyses showed that grape skin extract had a significantly greater phenol content (*p* < 0.01) than seed and pomace extracts (Table 1). The presence of phenolic compounds in wine byproducts has been associated with antioxidant and antidiabetic properties. In this sense, the significantly higher (*p* < 0.001) antioxidant capacity measured by ABTS of skin and seed extracts, as compared to that of grape pomace, could be associated to their greater phenol content. These results are in agreement with those of a previous study evaluating grape extracts on the cheese-making properties of milk [16]. 

Inhibition of α-glucosidase activity of grape skin and seed extracts was significantly higher (*p* < 0.001) than that of grape pomace (Table 1). IC50 of the control acarbose was 0.0045 mg/mL. IC50 of grape skin extract (0.30 ± 0.03 mg/mL) was similar to previously reported values from the Norton variety grape skin extract (0.38 mg/mL) [7]. However, IC50 of grape pomace (0.55 ± 0.06 mg/mL) and seed (0.36 ± 0.06 mg/mL) extracts were lower than those previously reported in red wine grape pomace of Cabernet Franc variety (1.63 mg/mL) [17] and muscadine seed extracts (1.53 mg/mL) [18]. This divergence may be due to differences in extraction methods, grape varieties or methods for assessing α-glucosidase inhibition. Previous studies have associated the inhibition of the enzymatic α-glucosidase activity of grapes with the presence of quercetin and ellagic acid in seeds, and anthocyanins and catechins from the skin and grape pomace [7,19]. Overall, wine byproducts showed strong α-glucosidase inhibition, which was higher than that reported in other natural inhibitors, such as green tea (IC50 = 2.04 mg/mL), oolong tea (IC50 = 2.33 mg/mL) or black tea (IC50 = 2.73 mg/mL) [20].

### 3.2. Application of Inulin-Type Fructans and Winery Byproduct Extracts in Yogurt 

#### 3.2.1. Yogurt Composition

TPC of control yogurts significantly increased with the addition of winery byproduct extracts (*p* < 0.001) (Table 2). The grape skin yogurts (Y-ISK and Y-SK) showed a significantly higher TPC than yogurts containing grape pomace and seed extracts (*p* < 0.001), which may be due to the higher TPC of the skin extract (Table 1). The addition of inulin and FOS did not modify the TPC in control and wine-extract yogurts (*p* > 0.05). 

Total lactose, protein and fat content in Y-IC did not differ (*p* > 0.05) from those of yogurts containing both inulin-type fructans and winery byproduct extracts (Y-IGP, Y-IS, Y-ISK) (Table 3). Furthermore, non-significant differences (*p* > 0.05) were found in the fatty acid profile between yogurt formulations. Levels of individual fatty acids were in accordance with previous analyses on the fatty acid composition of cow yogurts [21]. Palmitic acid (C16:0) was the most predominant fatty acid, followed by oleic acid (C18:1n9c), myristic acid (C14:0) and stearic acid (C18:0). The CLA content of the yogurts containing winery byproduct extracts, FOS and inulin (0.45–0.48%) was in accordance with those previously reported in cow milk yogurts (0.24–0.45%) [21]. The fatty acid profile of the developed yogurts showed low atherogenicity (2.95–2.97) and thrombogenicity (3.98–3.99) indexes, which are indicators to predict the risk of atherosclerosis and the tendency to form clots in the blood vessels [22]. The atherogenicity and thrombogenicity indexes of the studied yogurts were lower than those observed in yogurts from sheep milk made with increasing doses of inulin [23].

#### 3.2.2. Health-Promoting Properties

Yogurts containing winery byproduct extracts showed a significant increase (*p* < 0.001) in their antioxidant capacity, as compared to the control yogurts (Y-C, Y-IC) (Table 2). This may be associated with the TPC of the winery byproduct extracts. Y-SK and Y-ISK had greater antioxidant capacity measured by ABTS (*p* < 0.01) than yogurts containing grape pomace and seed extracts (Y-GP, Y-S and Y-IGP, Y-IS). This is in accordance with the TPC of the winery byproduct extracts, which was also significantly higher (*p* < 0.01) in the grape skin extract, when compared to the TPC of grape pomace and seed extracts (Table 1). Results of the TPC and consequent antioxidant capacity of yogurts containing winery byproduct extracts are in the range of those previously reported [24,25]. Adding inulin and FOS did not modify (*p* > 0.05) the antioxidant capacity measured in yogurts. 

To our knowledge, this is the first time that the in vitro inhibition of the enzymatic activity of α-glucosidase has been determined in yogurts containing winery byproduct extracts with inulin and FOS. Inhibition of α-glucosidase activity was significantly higher (*p* < 0.001) in yogurts containing grape skin and grape pomace extracts, as compared to the controls. These results suggest that the increase of inhibition of α-glucosidase activity may be due to the phenolic compounds present in the grape skins. Previous studies have shown that the inhibition of α-glucosidase activity from Tannat grape skin extract was partly due to the presence of cyanidins [26]. Anthocyanins, tannins, chlorogenic acid and other polyphenols can inhibit α-glucosidase activity [27] and could be present in the grape skin and grape pomace extracts, explaining the inhibition capacity of the yogurts containing these extracts (Y-GP, Y-SK, Y-IGP and Y-ISK). Although the grape seed extract showed a high inhibition of α-glucosidase activity (IC50 = 0.36 mg/mL) (Table 1), yogurts containing grape seed extract (Y-S, Y-IS) did not present a significant inhibition of the enzyme’s activity (*p* > 0.05), as compared to that of the control yogurts (Y-C, Y-IC). These results suggest that the observed inhibition of α-glucosidase activity in yogurts containing winery byproduct extracts may depend on both the profile of bioactive compounds and their molecular interactions with other components present in the food matrix, such as proteins. Such interactions have been described in bread fortified with grape skin extract [28]. In this study, the fortified bread showed a lower inhibition of the activity of α-glucosidase compared to that of the isolated grape skin ingredient, which was probably due to the formation of protein/polysaccharide/proanthocyanidin complexes.

Adding inulin and FOS in yogurt did not significantly modify the enzyme’s activity inhibition values (*p* > 0.05) (Table 2). Inulin and FOS have also been associated with an improvement of glucose regulation by the modification of satiety hormone response (PYY and GLP-1) [29]. Therefore, the combined antidiabetic mechanisms of winery byproduct extracts and inulin and FOS may provide a novel approach to improve glucose metabolism. 

#### 3.2.3. Technological Parameters and Shelf-Life Characterization

Characterization of pH, titratable acidity, moisture, syneresis, viability of starter bacteria and instrumental texture during 21 days of cold storage is shown in Table 4. The addition of winery byproduct extracts did not significantly (*p* > 0.05) modify the yogurt’s pH or titratable acidity, which is in accordance with previous results obtained in yogurts containing grape seed extracts [24]. However, other studies have found that the addition of grape byproducts to yogurt significantly lowered its pH [15,30,31]. Discrepancies between results may be due to differences in yogurt formulations, such as the quantity and intrinsic pH value of the grape extract or flour used [31], the metabolic activity of certain probiotic strains [25] and the interaction with other ingredients present in the formulation such as grape juice, sucrose, inulin and FOS [15,25]. The addition of inulin has been described to decrease the yogurts titratable acidity [23]. Therefore, inulin and FOS may help reduce post-acidification in yogurts containing winery byproduct extracts.

Y-IGP, Y-IS and Y-ISK showed a significantly syneresis increase (*p* < 0.05) during the last two weeks of storage, which is in line with the increased syneresis observed during storage in yogurts fortified with grape pomace flour [31]. According to the protein–polyphenol interaction model [32], the high syneresis values observed in yogurts fortified with winery byproduct extracts could be due to the great amount of polyphenols present in the food matrix. The incorporation of polyphenols increases the number of particle–particle junctions in the gel structure, leading to the shrinkage of the network and expulsion of interstitial liquid [33]. Moreover, previous studies using wine grape pomace in yogurt [31] have described that the timing of addition of the polyphenol-rich extracts can also affect yogurt syneresis. Greater syneresis rates were observed when the extracts were added to milk before fermentation than when they were added once the yogurt gel was formed. This could also explain the high syneresis values obtained in our study, as the winery byproduct extracts were added before milk fermentation. Further information is needed to understand the interactions of the three ingredients (inulin-type fructans, milk proteins and polyphenols) in the yogurt matrix.

The significantly higher syneresis rate of the control yogurt containing inulin and FOS (Y-IC) at day one after yogurt elaboration may be due to its higher pH value (4.75, Table 4). The coagulum obtained at a pH above 4.5 is presumably weaker than for a lower pH, which presents a firmer structure due to stronger protein–protein bonds [34]. Therefore, the resistance of the gel to the centrifugation force may have been lower, resulting in a higher syneresis value. Syneresis rates did not increase during storage, which may be due to the texture-improving properties that inulin confers to yogurts [5]. This is in accordance with previous research, in which the addition of up to 2% of inulin visibly reduced syneresis in low fat yogurts [35]. Scanning electron micrographs revealed that inulin formed elongated gelled structures that intermingled into the protein network of reduced milk-fat yogurts [36]. Increasing concentrations of inulin resulted in the formation of more secondary gelled structures between casein micelles aggregates, which fortified the yogurt network by acting as a water structuring agent.

Moisture content was significantly higher (*p* < 0.05) in grape skin yogurt (Y-ISK) than in the control (Y-IC) during storage. This was contrary to our expectations, as addition of the extracts increased the percentage of dry matter, reducing the amount of moisture. This deviant result in grape skin extracts could be due to the different interactions between grape skin polyphenols and other matrix components.

The instrumental texture parameters of functional yogurts are shown in Table 4. The addition of the winery byproduct extracts showed a tendency toward increasing the firmness and consistency parameters of yogurts (Y-IGP, Y-IS and Y-ISK), as compared to the control Y-IC. The firmness and consistency values of Y-IGP, Y-IS and Y-ISK were similar to those of Greek yogurt, probably due to the texturizing properties of inulin [4,37]. Regarding shelf life, previous studies have observed a significant increase in the firmness and consistency of yogurts containing grape pomace extract after 28 days of cold storage [25]. Although no significant differences (*p* > 0.05) were observed in the present study, during storage, firmness and consistency tended to increase slightly with time. 

Results of the microbiological analysis are shown in Table 4. Bacterial counts throughout the shelf life of the control and winery-byproduct-extract yogurts were higher than the minimum of 7 log CFU/g legally required in yogurt manufacture by the Codex Alimentarius. The initial and final *S. thermophilus* counts (9.05–9.18 and 9.00–9.16 log CFU/g, respectively) were significantly higher (*p* < 0.001) than those obtained for *L. delbrueckii* subsp. *bulgaricus* (5.56–5.89 and 4.96–5.11 log CFU/g, respectively). *S. thermophilus*, in general, survives well (>10^8^ cfu/mL) in fermented milk products during refrigerated storage [38], showing insignificant viability variations in yogurts containing apple pomace flour [39] or tea extracts during storage [40]. Lower counts of *L. delbrueckii* subsp. *bulgaricus* than *S. thermophilus* is a common observation in yogurt elaboration with commercial cultures of bacteria, which may be a strategy to minimize the acetic acid taste produced from the metabolism of *L. delbrueckii* subsp. *bulgaricus* [41]. 

The addition of winery byproduct extracts to yogurt did not significantly affect (*p* > 0.05) bacterial counts during storage, suggesting that the phenolic compounds of winery byproduct extracts did not affect the viability of starter bacteria. The presence of inulin and FOS may have affected the fermentation kinetics of the yogurts. Previous studies have shown that the addition of prebiotics, such as inulin, reduces the fermentation times of yogurt formation, as the dietary fiber acts as an additional source of carbohydrates for the probiotic bacteria [42]. The lower yogurt fermentation time could adversely affect the physicochemical and sensory attributes of the yogurt, as an accelerated fermentation may lead to the formation of a weak gel with large pores and greater syneresis [43]. Therefore, further studies are needed to elucidate the combined effect of winery byproduct extracts and inulin-type fructans on yogurt fermentation kinetics.

#### 3.2.4. Pilot Consumer Test

Consumers scored all yogurt formulations similarly (*p* > 0.05) in terms of smell, flavor, texture and overall acceptability (Table 5). Significant differences were only observed in appearance, which was higher in the control and grape skin yogurts (*p* < 0.05) (6.96 and 7.30, respectively) than in the grape pomace and seed yogurts (5.96 and 5.93, respectively). Consumers may have recognized the control as a conventional yogurt, and its familiarity could explain its high visual acceptability. The novel food color provided by the grape skin extract was also highly accepted, suggesting that color innovations in a purple palette for yogurts could be marketable. The influence of color on food preference has been extensively studied. Food coloring can influence flavor identification, perception and preferences, and it can even dominate other flavor sources of information, such as labelling and taste [44]. As the consumer test was conducted in blind conditions (no information on the composition of yogurts was provided to volunteers), the color–flavor associations made by consumers were unknown. 

In general, yogurt formulations obtained high overall liking scores, which ranged between 6.3 and 6.7, on a 9-point hedonic scale. However, several studies have reported a negative correlation between winery byproduct concentrations and overall yogurt acceptance [15,31]. Studies in which winery byproducts were added together with other ingredients significantly improved yogurt formulation. The addition of 5% sucrose [45], grape juice and sucrose [25], or a combination of oligofructose and purple grape juice [15], improved the overall acceptance of yogurts containing grape pomace and grape skin preparations. In this sense, we used inulin and FOS to improve the sensory properties and meet the desired fiber enrichment. Inulin has previously been described to improve yogurt texture and mouthfeel [4]. FOS are more soluble and sweeter than inulin, and can also improve mouthfeel [46]. The sweetening power (30–35%) and low caloric content (1–2 kcal/g) of FOS justified their use as a sucrose replacer. The dietary fiber content of the yogurts would also allow a “high in fiber” nutritional claim on the package. In this sense, an 80 g portion of the formulated yogurts would provide half the dietary fiber daily intake recommended by the EFSA.

## 4. Conclusions

This study showed that grape pomace, seed and skin byproduct extracts can be used together with inulin and FOS in the development of yogurt as sources of bioactive compounds and dietary fiber. Among the winery byproduct extracts, the grape skin extract presented a significant (*p* < 0.01) greater TPC, overall antioxidant capacity and inhibition of the α-glucosidase activity. The use of 5 mg/mL of grape skin extract conferred antioxidant and antidiabetic properties to the yogurt, which were not modified by the addition of inulin and FOS (*p* > 0.05). To our knowledge, this is the first time that the in vitro antidiabetic properties of the grape skin yogurt have been described. Yogurt formulations containing winery byproduct extracts and dietary fiber showed similar physicochemical, textural and microbiological properties during a 21-day shelf life. The consumer pilot test indicated that the grape skin yogurt’s appearance achieved the best acceptance score, suggesting that the grape skin extract could have a potential use as a colorant in dairy products. Therefore, we propose the yogurt containing grape skin extract, together with inulin and FOS, as a promising candidate for the development of health-promoting and sustainable yogurts. 

## Figures and Tables

**Table 1:** TPC, antioxidant capacity and antidiabetic properties of winery byproduct extracts.

Parameters	Grape Pomace	Seed	Skin
TPC (mg GAE/g extract)	278.07 ± 113.01 ^a^	437.50 ± 4.23 ^b^	502.04 ± 27.06 ^c^
*Antioxidant capacity*			
ABTS (mmol TE/g extract)	4.64 ± 0.17 ^a^	8.68 ± 0.51 ^b^	9.10 ± 0.50 ^b^
ORAC (mmol TE/g extract)	6.28 ± 0.74 ^a^	7.32 ± 0.48 ^a^	11.22 ± 0.76 ^b^
*Antidiabetic properties*			
α-Glucosidase inhibition (IC50 mg/mL)	0.55 ± 0.06 ^b^	0.36 ± 0.06 ^a^	0.30 ± 0.03 ^a^

Values represent mean ± standard deviation. Different letters are significantly different at *p* < 0.01; TPC, total phenolic content; GAE, gallic acid equivalents; TE, Trolox equivalents; ABTS, *2,2′-azino-bis (3-ethylbenzothiazoline-6-sulphonic acid);* ORAC, oxygen radical absorbance capacity.

**Table 2:** TPC, antioxidant capacity and antidiabetic properties of yogurts containing winery byproduct extracts with or without inulin-type fructans.

Parameters	Winery Byproduct Extracts				Winery Byproduct Extracts + Inulin-Type Fructans			
	Control (Y-C)	Grape Pomace (Y-GP)	Seed (Y-S)	Skin (Y-SK)	Control (Y-IC)	Grape Pomace (Y-IGP)	Seed (Y-IS)	Skin (Y-ISK)
TPC (mg GAE/g yogurt)	0.03 ± 0.00 ^a^	0.06 ± 0.00 ^b^	0.07 ± 0.01 ^b^	0.09 ± 0.00 ^c^	0.04 ± 0.00 ^a^	0.07 ± 0.01 ^b^	0.07 ± 0.00 ^b^	0.09 ± 0.00 ^c^
*Antioxidant capacity*								
ABTS (mmol TE/g yogurt)	0.31 ± 0.01 ^a^	0.97 ± 0.06 ^b^	1.16 ± 0.10 ^bc^	1.49 ± 0.08 ^d^	0.28 ± 0.01 ^a^	0.95 ± 0.07 ^b^	1.04 ± 0.07 ^b^	1.37 ± 0.10 ^cd^
ORAC (mmol TE/g yogurt)	4.32 ± 0.36 ^a^	5.50 ± 0.30 ^ab^	6.34 ± 1.10 ^ab^	7.69 ± 1.15 ^b^	4.19 ± 1.23 ^a^	6.24 ± 1.09 ^ab^	5.60 ± 1.35 ^ab^	7.84 ± 1.07 ^b^
*Antidiabetic properties*								
α-Glucosidase inhibition (%)	31.61 ± 3.26 ^a^	50.92 ± 1.70 ^b^	38.52 ± 5.87 ^a^	56.46 ± 2.31 ^b^	33.45 ± 3.35 ^a^	51.58 ± 1.15 ^b^	38.89 ± 1.34 ^a^	53.05 ± 0.44 ^b^

Values represent mean ± standard deviation. Different letters are significantly different at *p* < 0.05; α-glucosidase inhibition (%) at yogurt concentration of 4 g/mL; TPC, total phenolic content; GAE, gallic acid equivalents; TE, Trolox equivalents; ABTS, *2,2′-azino-bis (3-ethylbenzothiazoline-6-sulphonic acid);* ORAC, oxygen radical absorbance capacity.

**Table 3:** Total lactose, protein, fat and fatty acid content (g/100 g of FA methyl esters) of yogurts containing inulin-type fructans together with grape pomace, seed and skin extracts, at day one after yogurt manufacture.

Parameters	Winery Byproduct Extracts + Inulin-Type Fructans			
	Control (Y-IC)	Grape Pomace (Y-IGP)	Seed (Y-IS)	Skin (Y-ISK)
Lactose (%)	3.24 ± 0.08	3.20 ± 0.2	3.10 ± 0.19	3.35 ± 0.51
Total Protein (%)	2.78 ± 0.07	2.72 ± 0.10	2.69 ± 0.02	2.67 ± 0.08
Total Fat (%)	2.88 ± 0.18	2.73 ± 0.24	2.70 ± 0.22	2.63 ± 0.18
Fatty acid profile (g/100 g FA methyl esters)				
C6:0	0.99 ± 0.02	0.96 ± 0.05	0.97 ± 0.02	0.97 ± 0.03
C8:0	1.03 ± 0.01	1.02 ± 0.05	1.01 ± 0.04	1.00 ± 0.04
C10:0	2.84 ± 0.10	2.81 ± 0.06	2.81 ± 0.09	2.81 ± 0.11
C11:0	0.08 ± 0.00	0.08 ± 0.00	0.08 ± 0.01	0.08 ± 0.00
C12:0	3.59 ± 0.11	3.55 ± 0.08	3.54 ± 0.06	3.56 ± 0.15
C14:0	12.18 ± 0.05	12.10 ± 0.09	12.09 ± 0.06	12.14 ± 0.14
C14:1n5	1.13 ± 0.03	1.13 ± 0.05	1.13 ± 0.05	1.12 ± 0.04
C15:0	1.33 ± 0.07	1.33 ± 0.08	1.34 ± 0.08	1.33 ± 0.06
C16:0	35.86 ± 0.39	35.95 ± 0.08	35.95 ± 0.50	36.03 ± 0.31
C16:1n7	1.82 ± 0.07	1.84 ± 0.09	1.82 ± 0.07	1.83 ± 0.07
C17:0	0.62 ± 0.02	0.63 ± 0.02	0.63 ± 0.02	0.62 ± 0.03
C18:0	9.92 ± 0.27	10.08 ± 0.55	10.08 ± 0.42	10.07 ± 0.32
C18:1n7c	1.12 ± 0.05	0.97 ± 0.26	1.01 ± 0.25	1.02 ± 0.13
C18:1n9c	21.52 ± 0.07	21.72 ± 0.33	21.69 ± 0.09	21.70 ± 0.26
C18:2n6c	2.44 ± 0.09	2.54 ± 0.09	2.46 ± 0.12	2.47 ± 0.10
C18:2n6t	0.23 ± 0.01	0.23 ± 0.02	0.23 ± 0.01	0.23 ± 0.01
C18:3n3	0.46 ± 0.04	0.46 ± 0.03	0.45 ± 0.04	0.46 ± 0.02
C18:3n6	0.05 ± 0.01	0.05 ± 0.01	0.05 ± 0.02	0.06 ± 0.01
C20:0	0.15 ± 0.01	0.16 ± 0.03	0.14 ± 0.01	0.15 ± 0.01
C20:1n9	0.07 ± 0.01	0.07 ± 0.01	0.08 ± 0.01	0.07 ± 0.00
C20:4n6	0.20 ± 0.02	0.21 ± 0.01	0.20 ± 0.01	0.21 ± 0.00
C20:5n3	0.05 ± 0.01	0.04 ± 0.01	0.04 ± 0.01	0.04 ± 0.00
C21:0	0.15 ± 0.02	0.15 ± 0.01	0.15 ± 0.02	0.15 ± 0.00
C22:0	0.06 ± 0.00	0.06 ± 0.02	0.06 ± 0.00	0.05 ± 0.01
C22:5n3	0.07 ± 0.00	0.08 ± 0.02	0.08 ± 0.02	0.07 ± 0.00
CLA	0.45 ± 0.01	0.47 ± 0.03	0.48 ± 0.02	0.47 ± 0.01
SFA	68.78 ± 0.08	68.88 ± 0.56	68.85 ± 0.31	68.97 ± 0.35
MUFA	25.67 ± 0.07	25.73 ± 0.05	25.73 ± 0.23	25.74 ± 0.31
PUFA	3.95 ± 0.16	4.07 ± 0.10	4.00 ± 0.17	4.00 ± 0.13

SFA, saturated fatty acids; MUFA, monounsaturated fatty acids; PUFA, polyunsaturated fatty acids. Results are expressed as mean ± SD (*n* = 3).

**Table 4:** Evaluation of the shelf-life stability of yogurts formulated with inulin-type fructans, together with grape pomace, seed and skin extracts, during 21 days of cold storage: physicochemical parameters, viability of starter bacteria and instrumental texture analysis.

Parameters	Days	Control (Y-IC)	Grape Pomace (Y-IGP)	Seed (Y-IS)	Skin (Y-ISK)	Significance
pH	1	4.75 ± 0.05 ^aC^	4.59 ± 0.07 ^aC^	4.60 ± 0.12 ^aC^	4.68 ± 0.05 ^aC^	ns
	7	4.39 ± 0.05 ^aB^	4.37 ± 0.08 ^aB^	4.44 ± 0.03 ^aB^	4.42 ± 0.04 ^aB^	ns
	14	4.32 ± 0.04 ^aAB^	4.33 ± 0.08 ^aAB^	4.37 ± 0.03 ^aAB^	4.32 ± 0.04 ^aAB^	ns
	21	4.31 ± 0.03 ^aA^	4.29 ± 0.05 ^aA^	4.35 ± 0.04 ^aA^	4.35 ± 0.04 ^aA^	ns
	Significance	***	***	***	***	
Titratable acidity (g lactic acid/ 100 g yogurt)	1	0.57 ± 0.08 ^aA^	0.63 ± 0.04 ^aA^	0.62 ± 0.05 ^aA^	0.57 ± 0.05 ^aA^	ns
	7	0.67 ± 0.05 ^aB^	0.70 ± 0.03 ^aB^	0.67 ± 0.05 ^aB^	0.67 ± 0.05 ^aB^	ns
	14	0.74 ± 0.03 ^aC^	0.72 ± 0.03 ^aC^	0.71 ± 0.03 ^aC^	0.73 ± 0.01 ^aC^	ns
	21	0.76 ± 0.03 ^aC^	0.78 ± 0.04 ^aC^	0.75 ± 0.03 ^aC^	0.74 ± 0.03 ^aC^	ns
	Significance	***	***	***	***	
Moisture (%)	1	74.90 ± 0.31 ^aA^	75.53 ± 0.54 ^abA^	76.24 ± 0.52 ^abA^	76.50 ± 0.59 ^bA^	*
	7	75.34 ± 0.47 ^aA^	76.24 ± 0.52 ^abA^	76.31 ± 0.39 ^abA^	76.70 ± 0.36 ^bA^	*
	14	75.47 ± 0.47 ^aA^	76.27 ± 0.53 ^aA^	76.50 ± 0.96 ^aA^	76.56 ± 0.70 ^aA^	ns
	21	75.12 ± 0.44 ^aA^	76.02 ± 0.76 ^abA^	76.86 ± 0.49 ^bA^	76.64 ± 0.34 ^bA^	*
	Significance	ns	ns	ns	ns	
Syneresis (%)	1	47.59 ± 4.38 ^bB^	30.75 ± 2.34 ^aA^	42.59 ± 5.26 ^abA^	34.57 ± 4.18 ^aA^	*
	7	43.89 ± 6.69 ^aB^	35.23 ± 3.09 ^aA^	41.94 ± 7.73 ^aA^	34.94 ± 2.65 ^aA^	ns
	14	40.72 ± 7.50 ^aA^	49.17 ± 4.16 ^aB^	48.94 ± 3.87 ^aB^	46.32± 5.18 ^aB^	ns
	21	40.77± 5.45 ^aA^	49.15 ± 4.31 ^aB^	50.13 ± 2.54 ^aB^	51.59 ± 4.34 ^aB^	ns
	Significance	*	*	*	*	
*L. delbrueckii* subsp. *bulgaricus* (log cfu/g)	1	5.56 ± 0.19 ^aB^	5.65 ± 0.22 ^aB^	5.89 ± 0.20 ^aB^	5.75 ± 0.12 ^aB^	ns
	7	5.57 ± 0.18 ^aB^	5.65 ± 0.22 ^aB^	5.84 ± 0.25 ^aB^	5.81 ± 0.11 ^aB^	ns
	14	5.43 ± 0.27 ^aAB^	5.49 ± 0.18 ^aAB^	5.60 ± 0.22 ^aAB^	5.29 ± 0.59 ^aAB^	ns
	21	5.10 ± 0.21 ^aA^	5.11 ± 0.33 ^aA^	4.96 ± 0.42 ^aA^	5.02 ± 0.19 ^aA^	ns
	Significance	***	***	***	***	
*S. thermophilus* (log cfu/g)	1	9.07 ± 0.05 ^aA^	9.14 ± 0.07 ^aA^	9.05 ± 0.09 ^aA^	9.18 ± 0.18 ^aA^	ns
	7	9.28 ± 0.08 ^aA^	9.29 ± 0.29 ^aA^	9.00 ± 0.26 ^aA^	9.11 ± 0.16 ^aA^	ns
	14	9.11 ± 0.01 ^aA^	9.00 ± 0.14 ^aA^	8.98 ± 0.04 ^aA^	8.94 ± 0.12 ^aA^	ns
	21	9.00 ± 0.17 ^aA^	9.16 ± 0.08 ^aA^	9.03 ± 0.19 ^aA^	9.02 ± 0.02 ^aA^	ns
	Significance	ns	ns	ns	ns	
Firmness (N)	1	1.88 ± 0.44 ^aA^	2.66 ± 0.51 ^aA^	2.79 ± 0.23 ^aA^	2.59 ± 0.91 ^aA^	ns
	7	2.25 ± 0.15 ^aA^	2.99 ± 0.14 ^aA^	2.70 ± 0.36 ^aA^	2.93 ± 0.24 ^aA^	ns
	14	2.63 ± 0.22 ^aA^	2.71 ± 0.20 ^aA^	2.90 ± 0.52 ^aA^	3.01 ± 0.56 ^aA^	ns
	21	2.30 ± 0.34 ^aA^	2.69 ± 0.67 ^aA^	2.92 ± 0.96 ^aA^	3.04 ± 0.47 ^aA^	ns
	Significance	ns	ns	ns	ns	
Consistency (Ns)	1	16.3 ± 4.21 ^aA^	24.82 ± 5.21 ^aA^	25.12 ± 3.71 ^aA^	24.08 ± 8.87 ^aA^	ns
	7	20.04 ± 1.05 ^aA^	26.69 ± 2.88 ^aA^	23.23 ± 6.04 ^aA^	26.92 ± 3.39 ^aA^	ns
	14	24.19 ± 1.94 ^aA^	23.74 ± 3.95 ^aA^	26.17 ± 4.93 ^aA^	24.23 ± 2.77 ^aA^	ns
	21	20.87 ± 2.25 ^aA^	22.69 ± 5.52 ^aA^	27.11 ± 9.17 ^aA^	26.95 ± 4.1 ^aA^	ns
	Significance	ns	ns	ns	ns	

Superscript uppercase letters in each column indicate statistically significant differences during storage. Superscript lowercase letters in each row indicate statistically significant differences between yogurt samples.* *p* < 0.05; ** *p* < 0.01; *** *p* < 0.001; ns, not significant.

**Table 5:** Pilot consumer analysis (*n* = 30) of yogurts containing inulin-type fructans and winery byproduct extracts.

Attributes	Control (Y-IC)	Grape Pomace (Y-IGP)	Seed (Y-IS)	Skin (Y-ISK)
	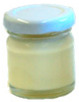	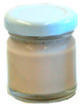	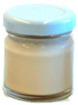	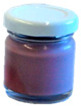
Appearance	6.96 ± 1.13 ^b^	5.96 ± 1.34 ^a^	5.93 ± 1.49 ^a^	7.30 ± 1.30 ^b^
Smell	6.30 ± 1.23 ^a^	5.96 ± 1.32 ^a^	5.44 ± 1.25 ^a^	5.78 ± 1.40 ^a^
Taste	6.67 ± 1.39 ^a^	6.52 ± 1.60 ^a^	6.48 ± 1.63 ^a^	5.89 ± 1.99 ^a^
Texture	7.07 ± 1.44 ^a^	7.04 ± 1.43 ^a^	6.74 ± 1.43 ^a^	6.85 ± 1.35 ^a^
Overall Acceptability	6.67 ± 1.36 ^a^	6.33 ± 1.52 ^a^	6.33 ± 1.41 ^a^	6.37 ± 1.64 ^a^

Data are expressed as mean ± standard deviation. Different letters indicate significant differences between yogurt samples (*p* < 0.05).

## Abbreviations

FOS, fructo-oligosaccharides; GAE, gallic acid equivalents; TPC, total polyphenol content.
